# Risk of systemic lupus erythematosus flares according to autoantibody positivity at the time of diagnosis

**DOI:** 10.1038/s41598-023-29772-w

**Published:** 2023-02-21

**Authors:** Oh Chan Kwon, Min-Chan Park

**Affiliations:** 1 Division of Rheumatology, Department of Internal Medicine, Yonsei University College of Medicine, Seoul, Korea; 2 Gangnam Severance Hospital, Yonsei University College of Medicine, 211 Eonjuro, Gangnam-Gu, Seoul, 06273 Korea

**Keywords:** Rheumatology, Risk factors

## Abstract

To estimate the risk of systemic lupus erythematosus (SLE) flares based on the autoantibody positivity at the time of SLE diagnosis. This retrospective cohort study included 228 patients with newly diagnosed SLE. Clinical characteristics including autoantibody positivity at the time of diagnosis of SLE were reviewed. Flares were defined as a new British Isles Lupus Assessment Group (BILAG) A score or BILAG B score for at least one organ system. Multivariable Cox regression analyses were performed to estimate the risk of flares according to autoantibody positivity. Anti-dsDNA, anti-Sm, anti-U1RNP, anti-Ro, and anti-La antibodies (Abs) were positive in 50.0%, 30.7%, 42.5%, 54.8%, and 22.4% of the patients, respectively. The incidence rate of flares was 28.2/100 person-years. Multivariable Cox regression analysis, adjusted for potential confounders, revealed that anti-dsDNA Ab positivity (adjusted hazard ratio [HR]: 1.46, *p* = 0.037) and anti-Sm Ab positivity (adjusted HR: 1.81, *p* = 0.004) at the time of diagnosis of SLE were associated with higher risk of flares. To better delineate the flare risk, patients were categorized as double-negative, single-positive, double-positive for anti-dsDNA and anti-Sm Abs. Compared with double-negativity, double-positivity (adjusted HR: 3.34, *p* < 0.001) was associated with higher risk of flares, while anti-dsDNA Ab single-positivity (adjusted HR: 1.11, *p* = 0.620) or anti-Sm Ab single-positivity (adjusted HR: 1.32, *p* = 0.270) was not associated with higher risk of flares. Patients who are double-positive for anti-dsDNA and anti-Sm Abs at the time of the diagnosis of SLE are at higher risk of flares and may benefit from stringent monitoring and early preventive treatment.

## Introduction

Systemic lupus erythematosus (SLE) is a systemic autoimmune disease characterized by excessive production of pathogenic autoantibodies against a range of autoantigens^1^. Of the pathogenic autoantibodies, autoantibodies against nuclear components (antinuclear antibodies, ANAs) are the hallmark of SLE that is detected in virtually all patients with SLE^2^. The particular antigens targeted by ANAs include double-stranded DNA (dsDNA) and extractable nuclear antigens (ENAs), such as Sm antigen, U1RNP antigen, Ro antigen, La antigen, etc^3,4^. Presence of autoantibodies against these particular autoantigens is known to be associated with specific manifestations of SLE: anti-dsDNA antibody (Ab) is associated with renal involvement and overall disease activity of SLE^5–7^; anti-Sm Ab is associated with renal involvement^8,9^; anti-U1RNP Ab is associated with Raynaud’s phenomenon and pulmonary hypertension^10,11^; and anti-Ro Ab and anti-La Ab are associated with cutaneous lupus, neonatal lupus, and sicca symptoms^5,12^.

During the course of the disease, SLE has periods of low disease activity, either by treatment or spontaneously, which are interspersed by periods of SLE flares^13^. The SLE flare events usually require intensified treatment^14^. An increased dose of glucocorticoids and/or immunosuppressants for the management of SLE flares is associated with adverse effects, including infections^15^. Moreover, the number of SLE flares is independently associated with an increased risk of damage accrual^16^. Therefore, it is important to identify patients with SLE at an increased risk of flare, preferably at early stages of the disease, as it can lead to optimized monitoring and preventive treatment. Although autoantibodies are known to play an important role in the pathogenesis of SLE^2^, association between their presence at the time of diagnosis and the risk of SLE flares is largely unknown. Studies have reported that increase in the titer of anti-dsDNA Ab during the disease course is associated with subsequent SLE flares^17–19^. However, it remains unclear whether the presence of autoantibodies including anti-dsDNA Ab at the time of diagnosis of SLE is associated with the risk of SLE flares during the disease course.

Here, we aimed to assess the risk of SLE flares according to the presence of anti-dsDNA Ab and anti-ENA Abs at the time of diagnosis of SLE.

## Methods

### Study population

Patients newly diagnosed with SLE between January 2009 and December 2019 at a tertiary referral hospital in Seoul, South Korea, were retrospectively included in the study. All patients fulfilled the 2012 Systemic Lupus International Collaborating Clinics (SLICC) classification criteria for SLE^20^. Patients who were followed up for less than a year were excluded. The retrospective observation period was from the date of SLE diagnosis to the last follow-up date, up to December 2021 or the date of first flare, whichever came first. The following data at the time of diagnosis of SLE were reviewed: age, sex, clinical manifestations of SLE, positivity of anti-dsDNA Ab, anti-Sm Ab, anti-U1RNP Ab, anti-Ro Ab, and anti-La Ab, levels of C3 and C4, and the SLE Disease Activity Index 2000 (SLEDAI-2 K)^21^. All patients who had symptoms or signs compatible to SLE and were positive for ANA were uniformly tested for anti-dsDNA, anti-Sm, anti-U1RNP, anti-Ro, and anti-La Abs. Therefore, there were no missing data on the positivity for each autoantibody. The positivity of anti-dsDNA Ab and anti-ENA Abs was tested using an automated fluoroimmunoassay analyzer (EliA; Phadia, Uppsala, Sweden). The use of the following medications during the observation period was reviewed: hydroxychloroquine, glucocorticoid, cyclophosphamide, mycophenolate mofetil, azathioprine, methotrexate, cyclosporin, and tacrolimus.

This study was approved by the Institutional Review Board of Gangnam Severance Hospital (No. 3–2020-0114). The requirement for informed consent was waived by the Institutional Review Board of Gangnam Severance Hospital due to the retrospective nature of this study. This study conformed the ethical guidelines laid out by the 1964 Helsinki declaration.

### Definition of flares

SLE flare was defined as (a) a new British Isles Lupus Assessment Group (BILAG) A score requiring high dose glucocorticoids (prednisolone > 20 mg per day or equivalent) and/or immunosuppressants or (b) a new BILAG B score requiring lower doses of glucocorticoids, antimalarials, or nonsteroidal anti-inflammatory drugs for at least one organ system, preceded by BILAG C–E scores^22^. Flares were further categorized as severe flares and mild-to-moderate flares. Severe flare was defined as the occurrence of a new BILAG A score, and mild-to-moderate flare was defined as the occurrence of a new BILAG B score.

### Statistical analysis

For the description of characteristics, continuous variables following normal distribution or non-normal distribution were expressed as mean (± standard deviation) or median (interquartile range), respectively. Categorical variables were expressed as numbers (%). One-way analysis of variance was used to compare continuous variables between four groups, and Bonferroni test was used for post-hoc analysis. Student’s t-test was used to compare continuous variables between two groups. Chi-square test or Fisher’s exact test was used to compare categorical variables between groups. Kaplan–Meier analysis was performed to visualize flare-free survival rates. Flare-free survival rates between different groups were compared using log-rank test. To assess whether the presence of anti-dsDNA Ab and anti-ENA Abs at the time of the diagnosis of SLE is associated with the risk of SLE flares, Cox proportional hazard regression analyses were used to estimate the hazard ratio (HR) with 95% confidence interval (95% CI). The proportional hazards assumption was tested by examining the log [− log (survival)] curves and Schoenfeld partial residuals; no relevant violations were identified. Univariable analyses using each autoantibody as the independent variable were performed, followed by multivariable analyses adjusted for age, sex, positivity of other autoantibodies, levels of complements, renal involvement, SLEDAI-2 K, and the use of hydroxychloroquine and immunosuppressants during the observation period. The analyses were first conducted using all flares (including both severe flare and mild-to-moderate flare) as the dependent variable, and then severe flare and mild-to-moderate flare were considered as the dependent variables to investigate whether a particular autoantibody is associated with the severity of the flare. P value < 0.05 was considered statistically significant. All analyses were conducted using the SPSS software (version 25.0; IBM Corporation, Armonk, NY, USA).

## Results

### Patient characteristics

A total of 228 patients with newly diagnosed SLE were included. The characteristics of the patients at the time of diagnosis of SLE are shown in Table 1. The mean age of the patients was 38.3 (± 14.9) years and majority of the patients were female (92.1%). The proportion of patients positive for anti-dsDNA Ab, anti-Sm Ab, anti-U1RNP Ab, anti-Ro Ab, and anti-La Ab were 50.0%, 30.7%, 42.5%, 54.8%, and 22.4%, respectively. The mean value of SLEDAI-2 K was 8.6 (± 5.6). During the observation period, hydroxychloroquine was used in 93.4% of the patients and glucocorticoid was used in all patients. In regard to immunosuppressants, cyclophosphamide, mycophenolate mofetil, azathioprine, methotrexate, cyclosporin, and tacrolimus were used in 9.6%, 19.7%, 23.7%, 7.9%, 3.9%, and 3.5% of the patients, respectively. None of the patients received rituximab or belimumab during the observation period.

**Table 1:** Characteristics of the patients with SLE.

Characteristics	N = 228
Age, years, mean (± SD)	38.3 (± 14.9)
Female, n (%)	210 (92.1)
Clinical manifestations, n (%)	
Mucocutaneous	126 (55.3)
Musculoskeletal	104 (45.6)
Hematologic	147 (64.5)
Renal	41 (18.0)
Serositis	21 (9.2)
Neuropsychiatric	17 (7.5)
Vasculitis	6 (2.6)
Autoantibody positivity, n (%)	
Anti-dsDNA Ab	114 (50.0)
Anti-Sm Ab	70 (30.7)
Anti-U1RNP Ab	97 (42.5)
Anti-Ro Ab	125 (54.8)
Anti-La Ab	51 (22.4)
C3, mg/dL, mean (± SD)	83.4 (± 33.0)
C4, mg/dL, mean (± SD)	18.9 (± 11.8)
SLEDAI-2 K, mean (± SD)	8.6 (± 5.6)
Medications used during observation period, n (%)	
Hydroxychloroquine	213 (93.4)
Glucocorticoid	218 (100.0)
Cyclophosphamide	22 (9.6)
Mycophenolate mofetil	45 (19.7)
Azathioprine	54 (23.7)
Methotrexate	18 (7.9)
Cyclosporin	9 (3.9)
Tacrolimus	8 (3.5)

*SLE* systemic lupus erythematosus; *ds* double-stranded; *Ab* antibody; *SLEDAI-2 K* systemic lupus erythematosus disease activity index 2000.

### Occurrence of flares

During a median observation period of 1.9 (0.8–3.7) years (total person-years of observation: 635.0 person-years), SLE flare events occurred in 179 (78.5%) patients. The number of flares per BILAG organ domain is shown in supplementary Table 1. The incidence rate of SLE flare was 28.2/100 person-years (Table 2). Severe flares occurred in 92 (40.4%) patients and mild-to-moderate flares occurred in 87 (38.2%) patients, corresponding to incidence rates of 14.5/100 person-years and 13.7/100 person-years, respectively.

**Table 2:** Summary of flare events.

	N = 228
Any flares	
Number of patients with flare events (%)	179 (78.5)
Incidence rate of flare/100 person-years	28.2
Severe flares	
Number of patients with flare events (%)	92 (40.4)
Incidence rate of flare/100 person-years	14.5
Mild-to-moderate flares	
Number of patients with flare events (%)	87 (38.2)
Incidence rate of flare/100 person-years	13.7

### Risk of flare according to the positivity of each autoantibody at the time of diagnosis of SLE

Univariable analysis revealed that anti-dsDNA Ab positivity (unadjusted HR: 1.47, 95% CI: 1.09–1.99, *p* = 0.011) and anti-Sm Ab positivity (unadjusted HR: 1.52, 95% CI: 1.12–2.07, *p* = 0.008) were associated with a higher risk of any flares. These associations remained statistically significant in the multivariable analysis adjusted for potential confounders (anti-dsDNA Ab positivity, adjusted HR: 1.46, 95% CI: 1.02–2.08, *p* = 0.037; and anti-Sm Ab positivity, adjusted HR: 1.81, 95% CI: 1.20–2.71, *p* = 0.004) (Table 3). The titers of anti-dsDNA Ab (adjusted HR: 1.001, 95% CI: 1.000–1.002, *p* = 0.004) and anti-Sm Ab (adjusted HR: 1.003, 95% CI: 1.001–1.005 *p* = 0.003) were also associated with an increased risk of any flares.

**Table 3:** Risk of flares according to the positivity of each autoantibody.

	Univariable analysis		Multivariable analysis^a^	
	Unadjusted HR (95% CI)	*P* value	Adjusted HR (95% CI)	*P* value
Any flares				
Anti-dsDNA Ab positivity	1.47 (1.09–1.99)	0.011	1.46 (1.02–2.08)	0.037
Anti-Sm Ab positivity	1.52 (1.12–2.07)	0.008	1.81 (1.20–2.71)	0.004
Anti-U1RNP Ab positivity	1.19 (0.89–1.60)	0.250	0.81 (0.55–1.19)	0.280
Anti-Ro Ab positivity	1.19 (0.88–1.61)	0.252	0.99 (0.67–1.46)	0.945
Anti-La Ab positivity	1.24 (0.89–1.74)	0.211	1.31 (0.85–2.02)	0.218
Severe flares				
Anti-dsDNA Ab positivity	2.05 (1.38–3.03)	< 0.001	1.63 (1.02–2.62)	0.041
Anti-Sm Ab positivity	1.73 (1.17–2.55)	0.006	1.55 (0.90–2.66)	0.116
Anti-U1RNP Ab positivity	1.64 (1.12–2.39)	0.011	1.22 (0.73–2.06)	0.452
Anti-Ro Ab positivity	1.13 (0.77–1.65)	0.528	0.97 (0.58–1.62)	0.907
Anti-La Ab positivity	1.04 (0.66–1.62)	0.879	1.36 (0.76–2.41)	0.301
Mild-to-moderate flares				
Anti-dsDNA Ab positivity	1.04 (0.72–1.51)	0.831	1.28 (0.82–2.00)	0.277
Anti-Sm Ab positivity	1.43 (0.97–2.11)	0.069	1.88 (1.14–3.09)	0.013
Anti-U1RNP Ab positivity	1.08 (0.75–1.57)	0.681	0.67 (0.42–1.07)	0.090
Anti-Ro Ab positivity	1.25 (0.86–1.82)	0.237	0.98 (0.59–1.63)	0.933
Anti-La Ab positivity	1.51 (1.01–2.25)	0.046	1.46 (0.86–2.47)	0.164

*HR* hazard ratio; *CI* confidence interval; *ds* double-stranded; *Ab* antibody.

^a^Multivariable analysis adjusted for age, sex, positivity of other autoantibodies, levels of complements, renal involvement, SLEDAI-2 K, and use of hydroxychloroquine and immunosuppressants during observation period.

Univariable analysis for the estimation of the risk of severe flares revealed that anti-dsDNA Ab positivity (unadjusted HR: 2.05, 95% CI: 1.38–3.03, *p* < 0.001), anti-Sm Ab positivity (unadjusted HR: 1.73, 95% CI: 1.17–2.55, *p* = 0.006), and anti-U1RNP Ab positivity (unadjusted HR: 1.64, 95% CI: 1.12–2.39, *p* = 0.011) were associated with a higher risk of severe flares. However, in the multivariable analysis, only the association with anti-dsDNA Ab positivity (adjusted HR: 1.63, 95% CI: 1.02–2.62, *p* = 0.041) remained statistically significant. The titer of anti-dsDNA Ab (adjusted HR: 1.002, 95% CI: 1.001–1.003, *p* < 0.001) was also associated with a higher risk of severe flares.

Univariable analysis for the estimation of the risk of mild-to-moderate flares revealed that anti-La Ab positivity (unadjusted HR: 1.51, 95% CI: 1.01–2.25, *p* = 0.046) was associated with a higher risk of mild-to-moderate flares. However, in the multivariable analysis, this association lost statistical significance (adjusted HR: 1.46, 95% CI: 0.86–2.47, *p* = 0.164), while anti-Sm Ab positivity (adjusted HR: 1.88, 95% CI: 1.14–3.09, *p* = 0.013) was newly identified as having statistically significant association with a higher risk of mild-to-moderate flares. The titer of anti-Sm Ab (adjusted HR: 1.003, 95% CI: 1.000–1.005, *p* = 0.019) was also associated with a higher risk of mild-to-moderate flares.

### Risk of flare according to anti-dsDNA Ab and anti-Sm Ab positivity

As anti-dsDNA Ab and anti-Sm Ab were identified as two autoantibodies associated with a higher risk of flares, we further categorized the patients according to their positivity status with respect to these two autoantibodies (double-negative, single-positive for anti-dsDNA Ab, single-positive for anti-Sm Ab, and double-positive) to better delineate the risk of flares. The comparison of characteristics between patients who were anti-dsDNA Ab ( +) vs. ( −), and anti-Sm Ab ( +) vs. ( −), and between patients with four levels of positivity for anti-dsDNA and anti-Sm Abs [( −)/( −) vs. ( +)/( −) vs. ( −)/( +) vs. ( +)/( +)] are summarized in Table 4. Patients who were anti-dsDNA Ab ( +) less commonly had mucocutaneous manifestations (43.0% vs. 67.5%, *p* < 0.001), more commonly had renal manifestations (28.1% vs. 7.9%, *p* < 0.001) and serositis (14.9% vs. 3.5%, *p* = 0.003), had lower C3 (71.8 ± 33.8 mg/dL vs. 95.2 ± 27.6 mg/dL, *p* < 0.001) and C4 (14.3 ± 10.2 mg/dL vs. 23.6 ± 11.5 mg/dL, *p* < 0.001) levels, and higher SLEDAI-2 K (11.1 ± 5.8 vs. 6.0 ± 4.1, *p* < 0.001), and more commonly received cyclophosphamide (14.9% vs 4.4%, *p* = 0.007), mycophenolate mofetil (29.8% vs. 9.6%, *p* < 0.001) and azathioprine (32.5% vs 14.9%, *p* = 0.002) than those who were anti-dsDNA Ab (-). Patients who were anti-Sm Ab ( +) were younger (33.7 ± 12.5 years vs. 40.3 ± 15.5 years, *p* = 0.002), more commonly had musculoskeletal manifestations (55.7% vs. 41.1%, *p* = 0.042) and serositis (15.7% vs. 6.3%, p = 0.024), more commonly positive for anti-U1RNP Ab (81.4% vs. 25.3%, *p* < 0.001) and anti-Ro Ab (70.0% vs. 48.1%, *p* = 0.002), and more commonly received cyclophosphamide (15.7% vs. 7.0%, *p* = 0.039) than those who were anti-Sm Ab ( −). In the comparison of characteristics according to the four levels of positivity for anti-dsDNA and anti-Sm Abs [( −)/( −) vs. ( +)/( −) vs. ( −)/( +) vs. ( +)/( +)], age (40.4 ± 15.0 years vs. 40.2 ± 16.0 years vs. 36.9 ± 11.9 years vs. 30.8 ± 12.5 years, *p* = 0.006), proportion of patients with mucocutaneous manifestations (66.3% vs. 42.3% vs. 70.6% vs. 44.4%, *p* = 0.003), renal manifestations (7.5% vs. 26.9% vs. 8.8% vs. 30.6%, *p* = 0.001), serositis (2.5% vs. 10.3% vs. 5.9% vs. 25.0%, *p* = 0.002), anti-U1RNP Ab positivity (30.0% vs. 20.5% vs. 70.6% vs. 91.7%, *p* < 0.001), and anti-Ro Ab positivity (51.2% vs. 44.9% vs. 61.8% vs. 77.8%, *p* = 0.008), C3 (96.2 ± 26.5 mg/dL vs. 75.3 ± 34.6 mg/dL vs. 93.1 ± 30.4 mg/dL vs. 64.1 ± 31.0 mg/dL, *p* < 0.001) and C4 (23.9 ± 11.3 mg/dL vs. 15.4 ± 9.6 mg/dL vs. 22.9 ± 11.9 mg/dL vs. 11.7 ± 11.2 mg/dL, *p* < 0.001) levels, SLEDAI-2 K (6.1 ± 4.4 vs. 10.6 ± 6.2 vs. 5.9 ± 3.3 vs. 12.0 ± 4.7, *p* < 0.001), and proportion of patients who received cyclophosphamide (2.5% vs. 11.5% vs. 8.8% vs. 22.2%, *p* = 0.006), mycophenolate mofetil (8.8% vs. 24.4% vs. 11.8% vs. 41.7%, *p* < 0.001), and azathioprine (11.3% vs. 30.8% vs. 23.5% vs. 36.1%, *p* = 0.007) were different between groups.

**Table 4:** Comparison of characteristics according to positivity of anti-dsDNA Ab and anti-Sm Ab.

	Anti-dsDNA Ab			Anti-Sm Ab			Four levels of positivity for anti-dsDNA and anti-Sm Abs				
	( +) (N = 114)	( −) (N = 114)	*P* value	( +) (N = 70)	( −) (N = 158)	*P* value	( −)/( −) (N = 80)	( +)/( −) (N = 78)	(-)/( +) (N = 34)	( +)/( +) (N = 36)	*P* value
Age, years, mean (± SD)	37.2 (± 15.6)	39.3 (± 14.2)	0.279	33.7 (± 12.5)	40.3 (± 15.5)	0.002	40.4 (± 15.0)	40.2 (± 16.0)	36.9 (± 11.9)	30.8 (± 12.5)	0.006^a^
Female, n (%)	104 (91.2)	106 (93.0)	0.623	63 (90.0)	147 (93.0)	0.433	76 (95.0)	71 (91.0)	30 (88.2)	33 (91.7)	0.574
Clinical manifestations, n (%)											
Mucocutaneous	49 (43.0)	77 (67.5)	< 0.001	40 (57.1)	86 (54.4)	0.704	53 (66.3)	33 (42.3)	24 (70.6)	16 (44.4)	0.003
Musculoskeletal	53 (46.5)	51 (44.7)	0.790	39 (55.7)	65 (41.1)	0.042	32 (40.0)	33 (42.3)	19 (55.9)	20 (55.6)	0.237
Hematologic	76 (66.7)	71 (62.3)	0.489	45 (64.3)	102 (64.6)	0.969	49 (61.3)	53 (67.9)	22 (64.7)	23 (63.9)	0.854
Renal	32 (28.1)	9 (7.9)	< 0.001	14 (20.0)	27 (17.1)	0.598	6 (7.5)	21 (26.9)	3 (8.8)	11 (30.6)	0.001
Serositis	17 (14.9)	4 (3.5)	0.003	11 (15.7)	10 (6.3)	0.024	2 (2.5)	8 (10.3)	2 (5.9)	9 (25.0)	0.002
Neuropsychiatric	10 (8.8)	7 (6.1)	0.449	4 (5.7)	13 (8.2)	0.505	7 (8.8)	6 (7.7)	0 (0.0)	4 (11.1)	0.257
Vasculitis	1 (0.9)	5 (4.4)	0.213	1 (1.4)	5 (3.2)	0.669	4 (5.0)	1 (1.3)	1 (2.9)	0 (0.0)	0.384
Autoantibody positivity, n (%)											
Anti-dsDNA Ab	114 (100.0)	0 (0.0)	N/A	36 (51.4)	78 (49.4)	0.774	0 (0.0)	78 (100.0)	0 (0.0)	36 (100.0)	< 0.001
Anti-Sm Ab	36 (31.6)	34 (29.8)	0.774	70 (100.0)	0 (0.0)	N/A	0 (0.0)	0 (0.0)	34 (100.0)	36 (100.0)	< 0.001
Anti-U1RNP Ab	49 (43.0)	48 (42.1)	0.893	57 (81.4)	40 (25.3)	< 0.001	24 (30.0)	16 (20.5)	24 (70.6)	33 (91.7)	< 0.001
Anti-Ro Ab	63 (55.3)	62 (54.4)	0.894	49 (70.0)	76 (48.1)	0.002	41 (51.2)	35 (44.9)	21 (61.8)	28 (77.8)	0.008
Anti-La Ab	26 (22.8)	25 (21.9)	0.874	14 (20.0)	37 (23.4)	0.568	18 (22.5)	19 (24.4)	7 (20.6)	7 (19.4)	0.936
C3, mg/dL, mean (± SD)	71.8 (± 33.8)	95.2 (± 27.6)	< 0.001	78.2 (± 33.8)	85.8 (± 32.4)	0.107	96.2 (± 26.5)	75.3 (± 34.6)	93.1 (± 30.4)	64.1 (± 31.0)	< 0.001^b^
C4, mg/dL, mean (± SD)	14.3 (± 10.2)	23.6 (± 11.5)	< 0.001	17.2 (± 12.8)	19.7 (± 11.3)	0.146	23.9 (± 11.3)	15.4 (± 9.6)	22.9 (± 11.9)	11.7 (± 11.2)	< 0.001^c^
SLEDAI-2 K, mean (± SD)	11.1 (± 5.8)	6.0 (± 4.1)	< 0.001	9.1 (± 5.1)	8.3 (± 5.8)	0.362	6.1 (± 4.4)	10.6 (± 6.2)	5.9 (± 3.3)	12.0 (± 4.7)	< 0.001^d^
Medications used during observation period, n (%)											
Hydroxychloroquine	104 (91.2)	109 (95.6)	0.182	65 (92.9)	148 (93.7)	0.779	77 (96.3)	71 (91.0)	32 (94.1)	33 (91.7)	0.543
Glucocorticoid	114 (100.0)	114 (100.0)	N/A	70 (100.0)	158 (100.0)	N/A					
Cyclophosphamide	17 (14.9)	5 (4.4)	0.007	11 (15.7)	11 (7.0)	0.039	2 (2.5)	9 (11.5)	3 (8.8)	8 (22.2)	0.006
Mycophenolate mofetil	34 (29.8)	11 (9.6)	< 0.001	19 (27.1)	26 (16.5)	0.061	7 (8.8)	19 (24.4)	4 (11.8)	15 (41.7)	< 0.001
Azathioprine	37 (32.5)	17 (14.9)	0.002	21 (30.0)	33 (20.9)	0.135	9 (11.3)	24 (30.8)	8 (23.5)	13 (36.1)	0.007
Methotrexate	11 (9.6)	7 (6.1)	0.326	8 (11.4)	10 (6.3)	0.188	5 (6.3)	5 (6.4)	2 (5.9)	6 (16.7)	0.278
Cyclosporin	6 (5.3)	3 (2.6)	0.499	5 (7.1)	4 (2.5)	0.137	2 (2.5)	2 (2.6)	1 (2.9)	4 (11.1)	0.141
Tacrolimus	7 (6.1)	1 (0.9)	0.066	3 (4.3)	5 (3.2)	0.704	1 (1.3)	4 (5.1)	0 (0.0)	3 (8.3)	0.131

*ds* double-stranded; *Ab* antibody; *SLEDAI-2 K* systemic lupus erythematosus disease activity index 2000.

^a^Significant differences in Bonferroni test: ( −)/( −) versus. ( +)/( +), *p* = 0.007; and ( +)/( −) versus. ( +)/( +), *p* = 0.010.

^b^Significant differences in Bonferroni test: ( −)/( −) versus. ( +)/( −), *p* < 0.001; ( −)/( −) versus. ( +)/( +), *p* < 0.001; ( +)/( −) versus. ( −)/( +), *p* = 0.033; and ( −)/( +) versus. ( +)/( +), *p* = 0.001.

^c^Significant differences in Bonferroni test: ( −)/( −) versus. ( +)/( −), *p* < 0.001; ( −)/( −) versus. ( +)/( +), *p* < 0.001; ( +)/( −) versus. ( −)/( +), *p* = 0.006; and ( −)/( +) versus. ( +)/( +), *p* < 0.001.

^d^Significant differences in Bonferroni test: ( −)/( −) versus. ( +)/( −), *p* < 0.001; ( −)/( −) versus. ( +)/( +), *p* < 0.001; ( +)/( −) vs. ( −)/( +), *p* < 0.001; and ( −)/( +) versus. ( +)/( +), *p* = 0.001.

**Figure 1:**
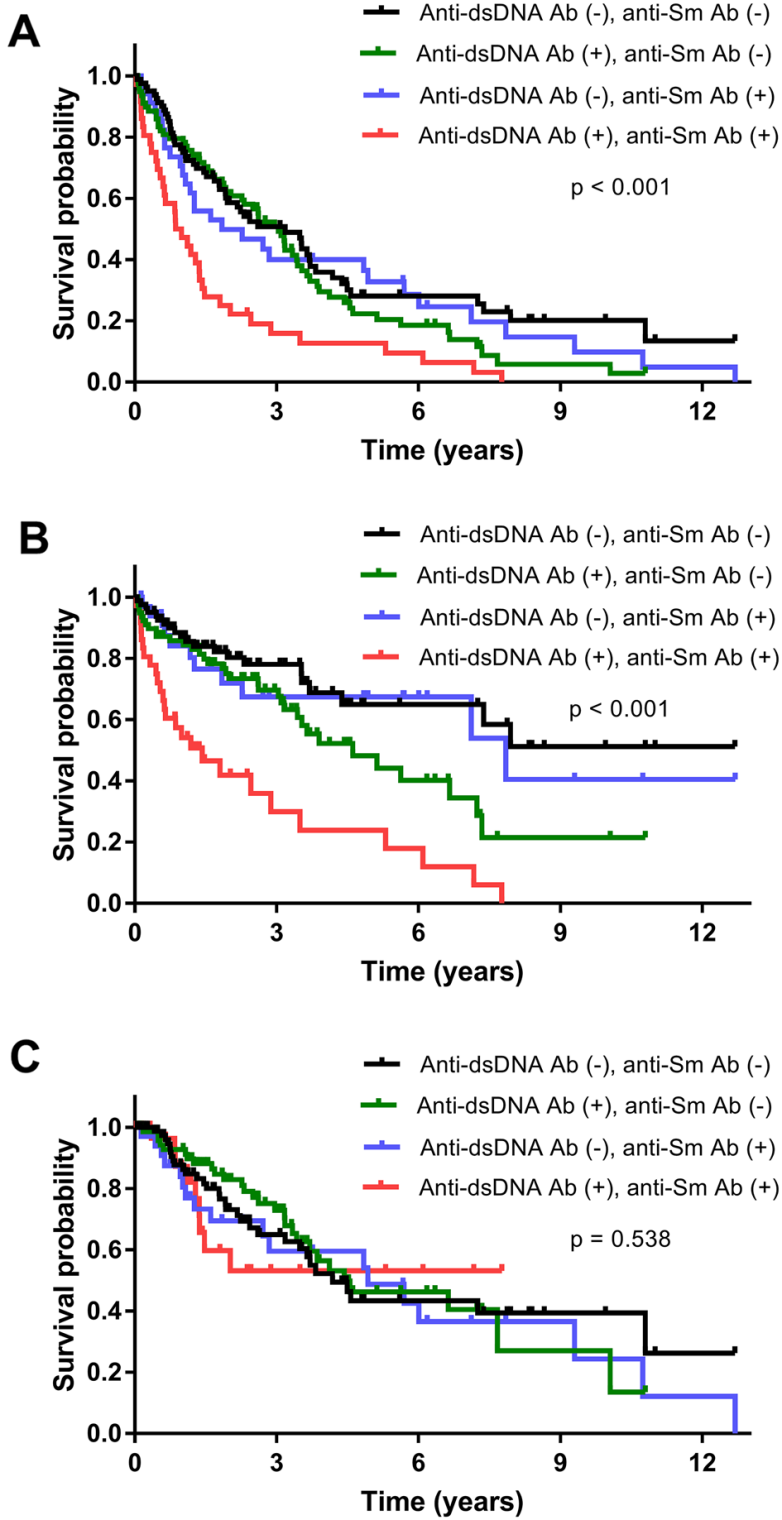
Flare-free survival curves according to the four levels of positivity for anti-dsDNA and anti-Sm Abs. (**A**) any flares, (**B**) severe flares, and (**C**) mild-to-moderate flares. ds—double-stranded; Ab—antibody.

The number of flare events and incidence rates of flares according to the positivity of anti-dsDNA and anti-Sm Abs are reported in Table 5. Occurrences of any flares and severe flares were higher in patients who were anti-dsDNA Ab ( +) than in those who were anti-dsDNA ( −), and in those who were anti-Sm Ab ( +) than in those who were anti-Sm Ab ( −). Among the four levels of positivity for anti-dsDNA and anti-Sm Abs, those who were double-positive for anti-dsDNA and anti-Sm Abs had the highest occurrence of any flares and severe flares, accounting for incidence rates of 59.3/100 person-years and 44.0/100 person-years, respectively. The flare-free survival rates according to the four levels of positivity for anti-dsDNA and anti-Sm Abs are shown in Fig. 1. Patients who were double-positive for anti-dsDNA Ab and anti-Sm Ab had the lowest survival rates for any flares (Fig. 1A, p < 0.001) and severe flares (Fig. 1B, p < 0.001), but not mild-to-moderate flares (Fig. 1C, p = 0.538).

**Table 5:** Flare events according to positivity of anti-dsDNA Ab and anti-Sm Ab.

	Anti-dsDNA Ab			Anti-Sm Ab			Four levels of positivity for anti-dsDNA and anti-Sm Abs				
	( +) (N = 114)	( −) (N = 114)	*P* value	( +) (N = 70)	( −) (N = 158)	*P* value	( −)/( −) (N = 80)	( +)/( −) (N = 78)	( −)/( +) (N = 34)	( +)/( +) (N = 36)	*P* value
Any flares											
Number of patients with flare events (%)	97 (85.1)	82 (71.9)	0.016	64 (91.4)	115 (72.8)	0.002	53 (66.3)	62 (79.5)	29 (85.3)	35 (97.2)	0.001
Incidence rate of flare/100 person-years	34.3	23.3		37.7	24.7		22.0	27.7	26.2	59.3	
Severe flares											
Number of patients with flare events (%)	60 (52.6)	32 (28.1)	< 0.001	37 (52.9)	55 (34.8)	0.010	21 (26.3)	34 (43.6)	11 (32.4)	26 (72.2)	< 0.001
Incidence rate of flare/100 person-years	21.2	9.1		21.8	11.8		8.7	15.2	9.9	44.0	
Mild-to-moderate flares											
Number of patients with flare events (%)	37 (32.5)	50 (43.9)	0.076	27 (38.6)	60 (38.0)	0.932	32 (40.0)	28 (35.9)	18 (52.9)	9 (25.0)	0.108
Incidence rate of flare/100 person-years	13.1	14.2		15.9	12.9		13.3	12.5	16.2	15.2	

*ds* double-stranded; *Ab* antibody.

For assessing the risk of flares according to the positivity of anti-dsDNA Ab and anti-Sm Ab, patients who were double-negative for anti-dsDNA Ab and anti-Sm Ab were considered as the reference group. Patients who were single-positive for anti-dsDNA Ab (any flare, adjusted HR: 1.11, 95% CI: 0.73–1.70, *p* = 0.620; severe flares, adjusted HR: 1.22, 95% CI: 0.69–2.16, *p* = 0.501; and mild-to-moderate flares, adjusted HR: 1.15, 95% CI: 0.68–1.93, *p* = 0.608) or single-positive for anti-Sm Ab (any flare, adjusted HR: 1.32, 95% CI: 0.80–2.18, *p* = 0.270; severe flares, adjusted HR: 1.08, 95% CI: 0.53–2.17, *p* = 0.839; and mild-to-moderate flares, adjusted HR: 1.69, 95% CI: 0.95–2.98, *p* = 0.072) did not exhibit significant association with higher risk of flares (Table 6). On the other hand, patients who were double-positive for anti-dsDNA Ab and anti-Sm Ab had statistically significant association with higher risk of flares (any flare, adjusted HR: 3.34, 95% CI: 1.85–6.01, *p* < 0.001; severe flares, adjusted HR: 2.93, 95% CI: 1.39–6.17, *p* = 0.005; and mild-to-moderate flares, adjusted HR: 2.80, 95% CI: 1.27–6.19, *p* = 0.011).

**Table 6:** Risk of flares according to the positivity of anti-dsDNA Ab and anti-Sm Ab.

	Univariable analysis		Multivariable analysis^a^	
	Unadjusted HR (95% CI)	*P* value	Adjusted HR (95% CI)	*P* value
Any flares				
Double-negative	1.00 (reference)		1.00 (reference)	
Single-positive for anti-dsDNA Ab	1.27 (0.88–1.84)	0.202	1.11 (0.73–1.70)	0.620
Single-positive for anti-Sm Ab	1.20 (0.76–1.89)	0.443	1.32 (0.80–2.18)	0.270
Double-positive	2.64 (1.71–4.07)	< 0.001	3.34 (1.85–6.01)	< 0.001
Severe flares				
Double-negative	1.00 (reference)		1.00 (reference)	
Single-positive for anti-dsDNA Ab	1.64 (1.00–2.68)	0.049	1.22 (0.69–2.16)	0.501
Single-positive for anti-Sm Ab	1.19 (0.63–2.23)	0.594	1.08 (0.53–2.17)	0.839
Double-positive	4.06 (2.34–6.95)	< 0.001	2.93 (1.39–6.17)	0.005
Mild-to-moderate flares				
Double-negative	1.00 (reference)		1.00 (reference)	
Single-positive for anti-dsDNA Ab	1.02 (0.65–1.60)	0.937	1.15 (0.68–1.93)	0.608
Single-positive for anti-Sm Ab	1.34 (0.80–2.25)	0.269	1.69 (0.95–2.98)	0.072
Double-positive	1.63 (0.91–2.94)	0.101	2.80 (1.27–6.19)	0.011

*ds* double-stranded; *Ab* antibody; *HR* hazard ratio; *CI* confidence interval.

^a^Multivariable analysis adjusted for age, sex, positivity of other autoantibodies, levels of complements, renal involvement, SLEDAI-2 K, and use of hydroxychloroquine and immunosuppressants during observation period.

## Discussion

It is well-known that disease activity fluctuates during the disease course of SLE^13,23^. A recent study using the SLICC cohort reported a flare incidence rate of 30/100 person-years for patients with SLE who were maintained on hydroxychloroquine^24^. In our study population, in which the majority of patients (93.4%) were receiving hydroxychloroquine, the incidence rate of flare was 28.2/100 person-years, which is similar to the incidence rate of the previous study^24^. It should be noted that the incidence rate of flare is high even in patients who are receiving hydroxychloroquine. This emphasizes the need to identify patients with a higher risk of flares at early stages of the disease, as these patients can benefit from closer monitoring and prompt treatment. In this study, we report that anti-dsDNA Ab and anti-Sm Ab double-positivity at the time of diagnosis of SLE is associated with a higher risk of flares during the disease course. This finding has clinical implication because it may help identify patients at a higher risk of flares early in the disease course.

Studies have reported multiple factors associated with the risk of SLE flares including age at the time of diagnosis^25,26^, sex^27^, ethnicity^27^, complements^27,28^, renal involvement^25,28^, disease activity^26,28^, and use of hydroxychloroquine^24,26,29,30^ and immunosuppressants^25,26^. However, previous studies investigating the risk factors of SLE flares did not evaluate the presence of anti-ENA Abs as a potential risk factor^24–30^. One recent study evaluated factors including autoantibody profile for association with the risk of SLE flares^31^ but the known potential confounders such as age, sex, ethnicity, complements, renal involvement, disease activity, and use of hydroxychloroquine and immunosuppressants were not adjusted in that study^31^. In the present study, we used multivariable analysis to adjust for potential confounders (ethnicity was not adjusted because all patients included in our study were Korean). Therefore, the associations between autoantibodies and risk of flares observed in our study seem independent of the potential confounders.

Multivariable analysis estimating the risk of flares according to the positivity of each autoantibody (Table 3) revealed statistically significant association of anti-dsDNA Ab (any flares, adjusted HR: 1.46; and severe flares, adjusted HR: 1.63) and anti-Sm Ab (any flares, adjusted HR: 1.81; and mild-to-moderate flares, adjusted HR: 1.88) with a higher risk of flares. However, when patients who were single-positive and double-positive for these autoantibodies were analyzed separately, the effect sizes were attenuated in patients who were single-positive for anti-dsDNA Ab (any flares, adjusted HR: 1.11; and severe flares, adjusted HR: 1.22) or single-positive for anti-Sm Ab (any flares, adjusted HR: 1.32; and mild-to-moderate flares, adjusted HR: 1.69) and lost statistical significance. That is, patients who were single-positive for anti-dsDNA Ab and those who were single-positive for anti-Sm Ab were not at a higher risk of flares. On the other hand, the effect sizes were accentuated in patients who were double-positive for anti-dsDNA Ab and anti-Sm Ab (any flares, adjusted HR: 3.34; severe flares, adjusted HR: 2.93; and mild-to-moderate flares, adjusted HR: 2.80). These results suggest that patients at higher risk of flares who may benefit from close monitoring are those who are double-positive for anti-dsDNA Ab and anti-Sm Ab, rather than those who are single-positive for each autoantibody.

With regard to other autoantibodies, positivity of anti-U1RNP Ab, anti-Ro Ab, and anti-La Ab was not associated with the risk of flares. Previous studies have shown that fluctuations of anti-U1RNP Ab, anti-Ro Ab, and anti-La Ab levels are not associated with flares in SLE^32,33^. Our finding adds to the previous knowledge that positivity of these autoantibodies at diagnosis is also not associated with the risk of flares.

Studies have reported that anti-dsDNA Ab level is associated with disease activity of SLE^5–7^. Other studies, although less robust, have reported that anti-Sm Ab level is associated with disease activity of SLE^34,35^. However, the association between positivity of anti-dsDNA Ab and anti-Sm Ab at the time of diagnosis of SLE and the risk of flares have not been studied. Our finding is novel in that we showed that anti-dsDNA Ab and anti-Sm Ab double-positivity as early as the onset of SLE is associated with a higher risk of flares during the disease course. Given that both anti-dsDNA Ab and anti-Sm Ab are associated with disease activity of SLE^5–7,34,35^, patients who are double-positive for these autoantibodies may have inherent high disease activity burden even if the disease activity is not yet overt at the time of diagnosis, and therefore, may have a higher chance of experiencing flares.

The present study has some limitations. First, variation of autoantibody titers over time was not taken into account. Some recent studies have reported that variation of laboratory markers over time is associated with flares in SLE^36,37^. One study reported that improvement of erythrocyte sedimentation rate, C-reactive protein, albumin, C3, proteinuria, urine red cells, and urine white cells over time is associated with lower risk of flares^36^. With regard to variation of anti-dsDNA Ab titers over time, studies have shown mixed results. One study reported no association between variation of anti-dsDNA Ab titers over time and risk of flares^36^, whereas another study reported significant decrease in anti-dsDNA Ab titers over time in patients who did not develop flares afterwards than in those who developed flares afterwards^37^. Further studies are needed to confirm the association between variations of autoantibody titers over time and risk of flares. Second, as this was a retrospective study, possibility of unmeasured confounding exists. For instance, we lack data on patients’ adherence to treatment, which could affect the risk of flares. Third, although we found interesting associations between autoantibody positivity at the time of diagnosis and future risk of flares, we lack mechanistic explanation for these associations. Fourth, the study population consists of patients of a single ethnicity. Considering that disease characteristics of SLE vary among patients with different ethnicities^38^, whether the present results may apply to other ethnic populations is unclear. Further prospective studies on various ethnic populations and mechanistic studies would be helpful in advancing our present findings.

In conclusion, we found that patients who are double-positive for anti-dsDNA Ab and anti-Sm Ab at the time of diagnosis of SLE have 3.34-fold higher risk of experiencing any flares during the disease course than those who are double-negative for anti-dsDNA Ab and anti-Sm Ab. In particular, anti-dsDNA Ab and anti-Sm Ab double-positivity at the time of diagnosis of SLE was associated with 2.93-fold higher risk of severe flares and 2.80-fold higher risk of mild-to-moderate flares. Therefore, patients who are double-positive for anti-dsDNA Ab and anti-Sm Ab at the time of diagnosis of SLE may benefit from stringent monitoring for flares and early preventive treatment, hopefully leading to reduced damage accrual.

## Supplementary Information

Supplementary Information.

## Data Availability

All data generated or analyzed during this study are included in this article.

## Abbreviations

SLE: Systemic lupus erythematosus
ANA: Antinuclear antibody
dsDNA: Double-stranded DNA
ENA: Extractable nuclear antigen
Ab: Antibody
SLICC: Systemic lupus international collaborating clinics
SLEDAI-2 K: Systemic lupus erythematosus disease activity index 2000
BILAG: British isles lupus assessment group
HR: Hazard ratio
CI: Confidence interval
