# Supplementary material for: Risk of systemic lupus erythematosus flares according to autoantibody positivity at the time of diagnosis
Source: Sci Rep. 2023 Feb 21;13:3068. doi: 10.1038/s41598-023-29772-w (PMC9945423; doi:10.1038/s41598-023-29772-w)
Supplement: Supplementary file 1 — Supplementary Information. [file 41598_2023_29772_MOESM1_ESM.docx]

Supplementary Table 1. Frequencies of flares per BILAG organ domain

| BILAG organ domain | N = 228 |
| --- | --- |
| Constitutional, n (%) | 72 (31.6) |
| Mucocutaneous, n (%) | 47 (20.6) |
| Neuropsychiatric, n (%) | 16 (7.0) |
| Musculoskeletal, n (%) | 41 (18.0) |
| Cardiorespiratory, n (%) | 4 (1.8) |
| Gastrointestinal, n (%) | 7 (3.1) |
| Ophthalmic, n (%) | 1 (0.4) |
| Renal, n (%) | 23 (10.1) |
| Hematological, n (%) | 20 (8.8) |

BILAG, British Isles Lupus Assessment Group
